# Impact of Preformed Donor-Specific Anti-Human Leukocyte Antigen Antibody C1q-Binding Ability on Kidney Allograft Outcome

**DOI:** 10.3389/fimmu.2017.01310

**Published:** 2017-10-31

**Authors:** Juan Molina, Ana Navas, María-Luisa Agüera, Cristian Rodelo-Haad, Corona Alonso, Alberto Rodríguez-Benot, Pedro Aljama, Rafael Solana

**Affiliations:** ^1^Maimonides Biomedical Research Institute of Cordoba (IMIBIC), Reina Sofia University Hospital, University of Cordoba, Cordoba, Spain; ^2^Department of Nephrology, Reina Sofia University Hospital, Cordoba, Spain; ^3^Department of Allergy and Immunology, Reina Sofia University Hospital, Cordoba, Spain; ^4^Department of Immunology, Infanta Cristina University Hospital, Badajoz, Spain

**Keywords:** allograft-loss risk, C1q-binding antibodies, kidney allograft survival, kidney transplantation, preformed anti-HLA antibodies, single antigen beads assay

## Abstract

The consolidation of single antigen beads (SAB-panIgG) assay in the detection of preformed anti-human leukocyte antigen (HLA) antibodies has improved transplantation success. However, its high sensitivity has limited the allograft allocation for sensitized patients, increasing their waiting time. A modification of the standard SAB-panIgG assay allows the detection of that subset of antibodies capable of binding C1q (SAB-C1q assay). However, the clinical usefulness of SAB-C1q assay for determining the unacceptable mismatches is under discussion. We retrospectively analyzed the impact of preformed donor-specific anti-HLA antibodies (DSA) according to the C1q-binding ability on allograft outcome, examining 389 single-kidney transplanted patients from deceased donors. Recipients with preformed C1q-binding DSA showed the lowest allograft survival up to 7 years (40.7%) compared to patients with preformed non-C1q-binding DSA (73.4%; *p* = 0.001) and without DSA (79.1%; *p* < 0.001). Allograft survival rate was similar between patients with preformed non-C1q-binding DSA and patients without preformed DSA (*p* = 0.403). Interestingly, among the high-mean fluorescence intensity DSA (≥10,000) population (*n* = 46), those patients whose DSA were further capable of binding C1q showed a poorer allograft outcome (38.4 vs. 68.9%; *p* = 0.041). Moreover, in our multivariate predictive model for assessing the risk of allograft loss, the presence of C1q-binding DSA (HR 4.012; CI 95% 2.326–6.919; *p* < 0.001) but not of non-C1q-binding DSA (HR 1.389; CI 95% 0.784–2.461; *p* = 0.260) remained an independent predictor after stratifying the DSA population according to the C1q-binding ability and adjusting the model for other pre-transplantation predictive factors including donor age, cold-ischemia time, and HLA-DR mismatches. In conclusion, the unacceptable mismatch definition according to the SAB-C1q assay would improve the risk stratification of allograft loss and increase the limited allograft allocation of highly sensitized patients, shortening their waiting time.

## Introduction

The presence of preformed antibodies against human leukocyte antigen (HLA), and specifically against those antigens expressed by the organ donor (donor-specific anti-HLA antibodies, DSA), is strongly associated with an increased risk of rejection and premature allograft failure (1). Against this background, the identification of antibody specificities in recipients awaiting solid organ transplantation has become a worldwide indispensable clinical practice to accurately assign their unacceptable HLA-antigen mismatches (2).

The complement-dependent cytotoxicity (CDC) assay has been considered the gold-standard method to detect circulating alloantibodies, since Patel and Terasaki demonstrated its usefulness for allograft allocation (3). Nowadays, despite the fact that CDC assay detects the presence of anti-HLA antibodies, solid-phase assays, like single antigen bead (SAB-panIgG) by Luminex technology, have been consolidated as the main standard methods, given their higher sensitivity to detect lower level of alloantibodies (4). Therefore, the actual definition of unacceptable alleles and the introduction of the non-invasive virtual cross-match (VXM) according to SAB-panIgG assay have improved the transplantation success, avoiding the allograft damage of anti-HLA antibodies not detectable by CDC. However, the higher sensitivity of SAB-panIgG assay has also increased the number of highly sensitized patients in transplantation waiting lists, making the graft allocation for these recipient candidates more difficult (5). As a result, many highly sensitized patients, with poor clinical prognosis, could die while waiting for a suitable donor.

Desensitization protocols emerged as an approach to reduce the levels of anti-HLA antibodies and expand transplantation possibilities of highly sensitized patients by immunomodulating the immune response (6). These strategies range from the use of plasmapheresis or intravenous immunoglobulin to monoclonal antibodies directed against CD20^+^ cells or against the C5 complement factor (7). Although desensitization to HLA may enable short-term success in incompatible transplantation, preventing the high rates of rejection and obtaining a durable reduction of anti-HLA antibody level remain a challenge (8). Hence, many efforts have been concurrently focused on understanding the true pathogenicity of anti-HLA antibodies.

Accordingly, the presence of preformed anti-HLA antibodies has been evaluated by the different available methods of detection, being the clinical relevance of anti-HLA antibodies detected by highly sensitive tests in a negative CDC context under discussion. The studies published to date have been controversial. Some groups have found no correlation between allograft failure and the presence of preformed DSA detected by SAB-panIgG assay (9–11). Other studies, however, have shown that the presence of these antibodies, undetected by other less sensitive tests, are associated with an increased risk of rejection and lower allograft survival (12–14).

The major pathway of antibody-induced cytotoxicity occurs subsequent to the antibody–antigen interaction, resulting in the activation of the classical complement pathway. The ability to activate the complement cascade is likely to be the key determinant of the pathogenic potential of many DSA (15). Hypothetically, antibodies with the ability to activate the complement cascade, among all antibodies detected by SAB-panIgG assay, could be more injurious to the allograft than those incapable of activating it (16, 17).

A new highly sensitive test has been developed to detect only the subset of anti-HLA antibodies capable of binding the first component of the human complement cascade, C1q (SAB-C1q assay) (18). Although information regarding SAB-C1q test results is still scarce, initial studies showed a high correlation between the presence of preformed C1q-binding DSA with early acute antibody-mediated rejection (AMR) and C4d staining in allograft biopsies (19, 20), thus supporting the general principle that antibodies capable of activating the complement cascade are the main antibodies involved in humoral rejection and allograft failure.

Given this possibility, our aim was to evaluate the clinical usefulness of SAB-C1q in the definition of immunological risk groups before transplantation. For this purpose, we retrospectively analyzed the impact of preformed DSA on allograft survival and allograft-loss risk in a single-kidney transplanted cohort, according to the C1q-binding ability.

## Materials and Methods

### Patient Selection

A total of 389 local single-kidney transplanted patients from local-deceased donors between January 1995 and October 2009 at Reina Sofia University Hospital (Cordoba, Spain) with available serum samples adequate for Luminex analysis were included in this study. All transplants were ABO group compatible. A negative T-cell and B-cell CDC cross-match in pre-transplantation neat-serum was required for all recipients. Triple maintenance immunosuppression was variable, but all transplanted recipients received a calcineurine inhibitor (cyclosporine or tacrolimus) combined with a DNA synthesis inhibitor (azathioprine or mycophenolate mofetil) and low-dose of steroids. Exceptionally, some included patients were treated with rapamycin combined with tacrolimus and prednisone. Since 1998, high immunological risk patients [panel reactive antibody (PRA) >80%, re-transplant with rejection as failure of the first graft] were induced with a polyclonal anti-human thymocyte immunoglobulin (thymoglobulin) for the first 4–7 days. Acute rejections were treated with steroids bolus for three consecutive days; steroid-resistant rejections were usually treated with OKT3 and since 1998, with thymoglobulin. No desensitization protocols were implemented to any recipients, since they are only offered to patients receiving a living-donor transplant according to guidelines followed by our center. The follow-up time was 7 years. The study was approved by the Ethics Committee of the Reina Sofia University Hospital (ref. 2465).

### Donor HLA Typing

All local donors were genotyped for the HLA-A, -B, -DRB1, and -DRB3/4/5 loci using a polymerase chain reaction sequence-specific oligonucleotide system (Dynal Reli SSO Test, Invitrogen Corporation). For all kidney transplanted patients with preformed anti-HLA-DQ antibodies detected by SAB-panIgG assay, donor’s HLA-DQB1 locus was retrospectively typed by molecular biology (Innolipa HLA-DQB1 typing kit; Innogenetics, Belgium).

### Detection and Characterization of Donor-Specific Antibodies

Neat pre-transplant serum samples from the 389 patients included, preserved at −20°C, were retrospectively screened using first the Luminex Mixed Screen assay (LABScreen Mixed I/II One Lambda Inc.). Then, patients with a positive screen (normalized background ratio ≥1.5) were characterized for anti-HLA antibody specificities (Class I and/or Class II) using SAB-panIgG assay (LABScreen single antigen beads, One Lambda Inc.). Samples were analyzed on a Luminex platform (LABScan 100) using Luminex 100 IS version 2.3 as data acquisition software and Fusion 3.0 program (One Lambda) as analysis software. Then, using the information on donor HLA typing, a VXM was performed. Positive VXM was considered when detecting an antibody in the recipient’s neat-serum against HLA-A, -B, -DRB1, -DRB3/4/5, or DQB1 donor’s molecules. This antibody was defined as DSA. Since no data for donors’ HLA-Cw and HLA-DP were available, anti-Cw and anti-DP antibodies were not considered in this study. In addition, patients’ sera with positive VXM by SAB-panIgG assay were analyzed by SAB-C1q assay (One Lambda Inc.) to detect complement-binding antibodies. Positive antibodies against a donor HLA antigen detected in this last test were considered C1q-binding DSA.

A cutoff for positive reactions was set at a baseline mean fluorescence intensity (MFI) value of ≥1,000 in the standard SAB-panIgG and at a baseline MFI value of ≥500 in the SAB-C1q assay. Antibodies with a baseline MFI value of ≥10,000 were considered high-MFI antibodies. All assays were performed according to the manufacturer’s instructions. The methodology of this study is illustrated in Figure S1 in Supplementary Material.

### Statistical Analysis

Patients’ characteristics were summarized using mean and SDs for the description of continuous variables, and total number and percentage for the description of non-continuous variables. The Student’s *t*-test was used to compare parametric quantitative data, while the Mann–Whitney *U* test was used to compare non-parametric quantitative data. The χ^2^ test, or Fisher’s *F* when required, was used to compare qualitative data. The Kolmogorov–Smirnov test was used as normality test. Pearson’s correlation was used to determine the association between high-strength antibodies and C1q-binding ability.

Allograft survival was analyzed since the time of transplantation up to 7 years with kidney allograft loss as the event of interest. Allograft loss was defined as return to dialysis. Data on graft survival were censored at the time of death. Kidney allograft survivals were plotted on Kaplan–Meier curves and compared according to the preformed anti-HLA antibody status using the log-rank test. The rejection incidence was not evaluated due to the heterogeneity in the diagnostic criteria throughout the study period.

Multivariate Cox regression was used to quantify hazard ratio(s) (HR) and 95% confidence intervals (CI) for kidney allograft loss. Collinearity tests were performed to ensure the independence of predictive and confounding variables. Receiver operator characteristic curves and area under the curve (AUC) were used to study models’ characteristics.

*p* Values lower than 0.05 were regarded as statistically significant.

## Results

### Patients’ Characteristics

The entire population (*n* = 389) was stratified into two groups according to the presence or absence of preformed DSA retrospectively detected by the standard SAB-panIgG assay. The DSA+ group comprised 92 (23.7%) patients who tested positive for the presence of preformed DSA, whereas the DSA− group included 297 (76.3%) patients who were negative. The clinical and immunological characteristics of both groups are shown in Table 1. When analyzing classical sensitization pathways against HLA molecules, a higher percentage of females (58.7 vs. 29.3%; *p* < 0.001), blood-transfused patients (71.7 vs. 38.7%; *p* < 0.001), and re-transplanted patients (41.3 vs. 6.4%; *p* < 0.001) were found in the DSA+ group compared to the DSA− group. Regarding the increased risk for developing anti-HLA antibodies, patients belonging to the DSA+ group had a higher PRA by CDC at time of transplantation (21.7 vs. 2.2; *p* < 0.001). Moreover, probably due to the difficulty of finding a suitable donor for these patients, their waiting time (years) was longer (8.0 vs. 3.8; *p* < 0.001). No statistically significant differences were found with regard to the other studied characteristics shown in Table 1.

**Table 1:** Clinical and immunological patient characteristics according to the donor-specific anti-HLA antibody (DSA) status at time of transplantation.

	Pre-transplantation anti-human leukocyte antigen (HLA) antibody status							
	DSA−			DSA+				*p*^a^	
	Anti-HLA− (*n* = 238)	Anti-HLA+/non-DSA (*n* = 59)	Total cohort (*n* = 297)	DSA+/C1q− (*n* = 62)	DSA+/C1q+ (*n* = 30)	*p*^b^	Total cohort (*n* = 92)		
**Donors**								

Age, mean (SD)	50.6 (17.1)	45.9 (17.3)	49.7 (17.2)	44.3 (17.9)	49.7 (20.2)	0.197	46.1 (18.8)	0.084
Cold-ischemia time (h), mean (SD)	17.8 (7.2)	17.2 (7.3)	17.7 (7.2)	17.2 (7.7)	18.9 (7.0)	0.307	17.8 (7.5)	0.927

**Recipients**								

Age, mean (SD)	49.1 (13.7)	47.1 (12.6)	48.7 (13.5)	45.6 (13.3)	48.8 (15.3)	0.305	46.7 (14.0)	0.219
Females, *n* (%)	67 (28.2)	20 (33.9)	87 (29.3)	35 (56.5)	19 (63.3)	0.530	54 (58.7)	<0.001
Re-transplanted patients, *n* (%)	8 (3.4)	11 (18.6)	19 (6.4)	21 (33.9)	17 (56.7)	0.037	38 (41.3)	<0.001
Blood-transfused patients, *n* (%)	82 (34.4)	34 (57.6)	115 (38.7)	46 (74.2)	20 (66.7)	0.452	66 (71.7)	<0.001
Time on waiting list (years), mean (SD)	3.3 (3.6)	5.6 (4.7)	3.8 (4.0)	8.5 (7.0)	7.0 (5.3)	0.317	8.0 (6.5)	<0.001
HLA-A, -B, -DR mismatches, mean (SD)	3.2 (1.3)	2.9 (1.0)	3.2 (1.2)	3.1 (1.1)	3.6 (1.1)	0.089	3.2 (1.1)	0.474
Anti-calcineurin drugs						0.869		0.971
Tacrolimus	132 (55.5)	32 (54.2)	164 (55.2)	34 (54.8)	17 (56.7)		51 (55.4)	
Cyclosporine	106 (44.5)	27 (45.8)	133 (44.8)	28 (45.2)	13 (43.3)		41 (44.6)	
Maintenance immunosuppressant triple therapy^c^						0.874		0.979
A, *n* (%)	164 (68.9)	36 (61.0)	200 (67.3)	42 (67.7)	21 (70.0)		63 (68.5)	
B, *n* (%)	59 (24.8)	21 (35.6)	80 (26.9)	17 (27.4)	7 (23.3)		24 (26.1)	
C, *n* (%)	15 (6.3)	2 (3.4)	17 (5.7)	3 (4.8)	2 (6.7)		5 (5.4)	
Pre-transplantation anti-HLA antibodies						n/c^d^		n/c^d^
Non-antibodies, *n* (%)	238		238 (80.1)	–	–			
Class I, *n* (%)	–	42 (71.2)	42 (14.1)	24 (38.7)	3 (10.0)		27 (29.3)	
Class II, *n* (%)	–	3 (5.1)	3 (1.0)	12 (19.4)	3 (10.0)		15 (16.3)	
Class I and II, *n* (%)	–	14 (23.7)	14 (4.7)	26 (41.9)	24 (80.0)		50 (54.3)	
Pre-transplantation^e^ PRA by CDC, mean (SD)	–	5.5 (13.4)	2.2 (9.6)	14.5 (24.2)	36.3 (36.9)	0.005	21.7 (30.5)	<0.001
Pre-transplantation^f^ cPRA, mean (SD)	–	39.4 (31.3)	7.8 (21.0)	81.1 (26.4)	97.7 (3.3)	<0.001	86.5 (23.1)	<0.001
Preformed DSA						0.253		
Against Class I, *n* (%)	–	–	–	42 (67.7)	16 (63.0)			
Against Class II, *n* (%)	–	–	–	18 (29.0)	11 (31.5)			
Against Class I and II, *n* (%)	–	–	–	2 (3.3)	3 (5.4)			

*^a^*p* value calculated for the comparison between DSA− (*n* = 297) and DSA+ (*n* = 92) groups*.

*^b^*p* value calculated for the comparison between DSA+/C1q− (*n* = 62) and DSA+/C1q+ (*n* = 30) groups*.

*^c^Triple immunosuppressant therapy consisted of calcineurine inhibitor + mycophenolate mofetil + corticosteroids (A); calcineurine inhibitor + azathioprine + corticosteroids (B); calcineurine inhibitor + mTor inhibitor + corticosteroids (C)*.

*^d^Non-compared characteristics*.

*^e^Pre-transplantation panel reactive antibody (PRA) value at time of transplantation, calculated by complement-dependent cytotoxicity (CDC) assay*.

*^f^Calculated panel reactive antibody (cPRA) value at time of transplantation, retrospectively calculated according to unacceptable antigens detected by SAB-panIgG assay using OPTN database*.

The 92 patients with preformed DSA were further stratified according to the DSA C1q-binding ability. Sixty-two (67.4%) DSA+ patients tested negative in SAB-C1q assay. These patients comprised the non-C1q-binding DSA group (DSA+/C1q−). The other 30 (32.6%) patients testing positive in SAB-C1q assay comprised the C1q-binding-DSA group (DSA+/C1q+). Table 1 also shows the clinical and immunological characteristics of both groups. We found a higher percentage of re-transplanted patients in the DSA+/C1q+ group compared to the DSA+/C1q− group (56.7 vs. 33.9%; *p* = 0.037). Probably as a consequence of previous transplants, which is the main sensitization pathway against HLA molecules (21), those patients had a significantly higher PRA by CDC and calculated PRA by SAB-panIgG assay (36.3 vs. 14.5; *p* = 0.005 and 97.7 vs. 81.1; *p* < 0.001). No statistically significant differences were found with regard to the other studied characteristics.

### Kidney Allograft Survival

**Figure 1:**
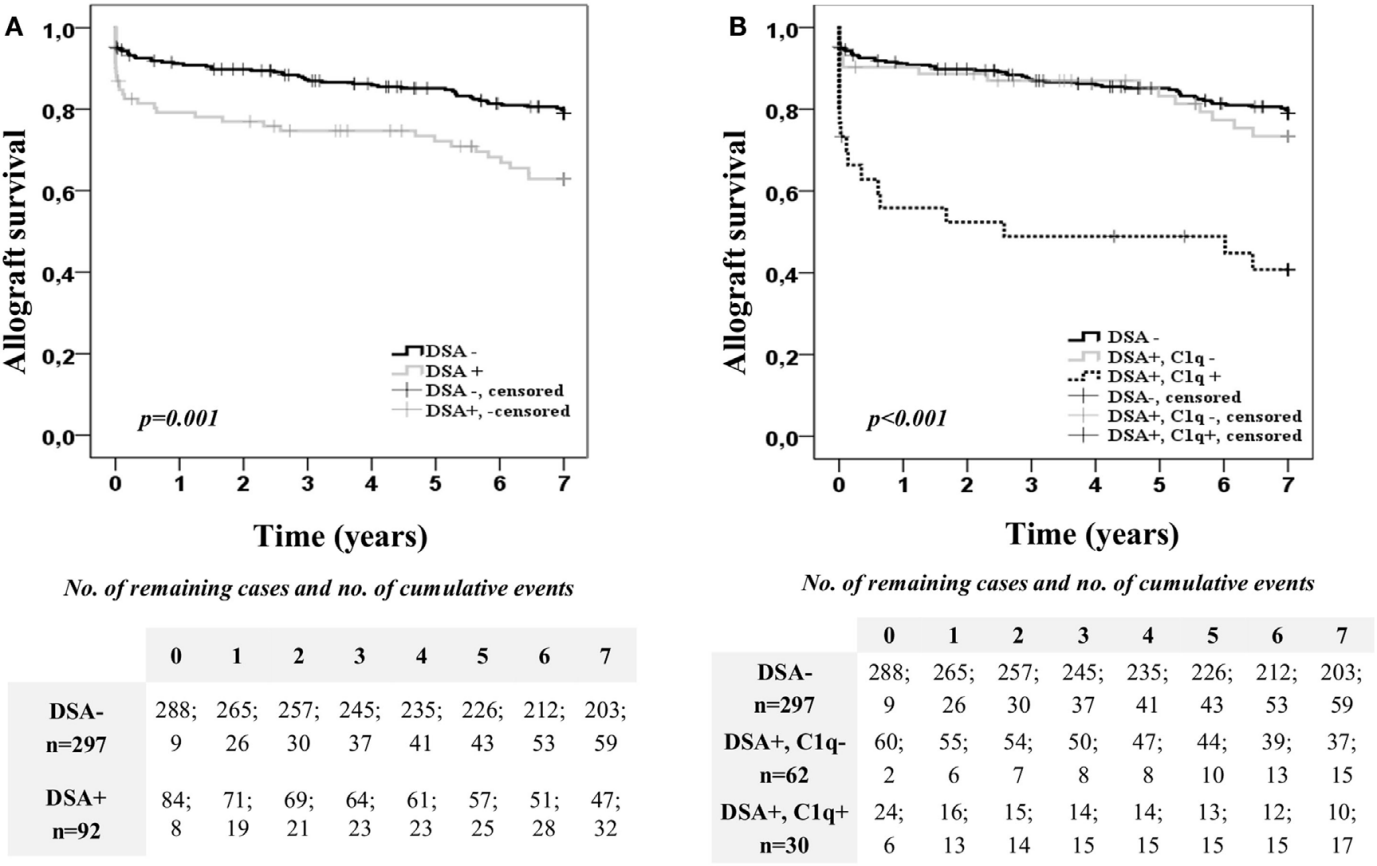
Allograft survival of the 389 single-kidney transplanted patients according to the donor-specific anti-HLA antibody (DSA) status at time of transplantation. Kaplan–Meier curves for allograft survival up to 7 years, stratified by the presence or absence of preformed DSA **(A)** and the DSA C1q-binding ability **(B)**. Curves were compared using the log-rank test.

Kaplan–Meier curves for kidney allograft survival according to the presence or absence of preformed DSA at time of transplantation are shown in Figure 1A. Patients with preformed DSA had significantly worse 7-year allograft survival than patients without preformed DSA (62.9 vs. 79.1%; *p* = 0.001). However, when the population with preformed DSA was categorized according to the DSA C1q-binding ability (Figure 1B), patients with preformed C1q-binding DSA had significantly the worst allograft survival among the study population. Thus, at the end of the follow-up time, only the 40.7% of patients with preformed C1q-binding DSA maintained their allograft functioning, whereas allograft function was maintained in the 73.4% of patients with preformed non-C1q-binding DSA (*p* = 0.001) and in the 79.1% of patients without preformed DSA (*p* < 0.001). Interestingly, 7-year allograft survival rate was similar between patients with preformed non-C1q-binding DSA and patients without preformed DSA (*p* = 0.403). When we examined allograft survival in the C1q-binding DSA population according to the presence of preformed DSA against Class I and/or Class II HLA molecules (Figure S2A in Supplementary Material), we did not find differences (*p* = 0.862).

**Figure 2:**
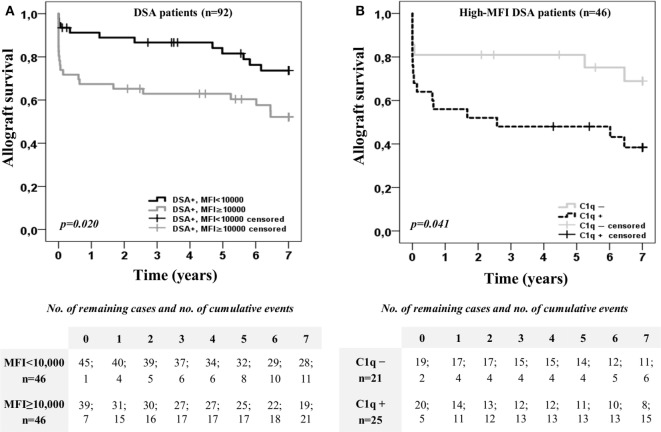
Kaplan–Meier curves for allograft survival up to 7 years of the 92 patients with donor-specific anti-HLA antibodies (DSA), categorized according to the presence or absence of high-mean fluorescence intensity (MFI) DSA (MFI ≥10,000) at time of transplantation **(A)**, and after stratifying high-MFI DSA group (*n* = 46) according to the C1q-binding ability **(B)**. Curves were compared using the log-rank test.

Furthermore, we explored the impact on allograft outcome of the C1q-binding ability considering also the DSA MFI value obtained by the standard SAB-panIgG (Figure 2). For this purpose, the DSA population was first categorized according to the presence or absence of high-MFI DSA (MFI ≥10,000) at time of transplantation. Kaplan–Meier curves for allograft survival according to the presence or absence of high-MFI DSA are plotted in Figure 2A. As expected, patients with preformed high-MFI DSA had lower allograft survival rate than the other DSA recipients (52.1 vs. 73.7%; *p* < 0.020). Then, we analyzed allograft survival up to 7 years of the high-MFI DSA group (*n* = 46), which was stratified according to the DSA C1q-binding ability (Figure 2B). Interestingly, patients with C1q-binding DSA showed a poorer allograft survival compared to patients with non-C1q-binding DSA (38.4 vs. 68.9%; *p* = 0.041), despite the fact that preformed DSA of both groups had a high-MFI value. No differences were found (*p* = 0.988) when high-MFI C1q-binding DSA were stratified according to the type of HLA molecules (Class I and/or Class II) against which they were directed (Figure S2B in Supplementary Material).

### Risk of Allograft Loss

We studied the association of clinical and immunological factors with allograft loss in a univariate analysis by Cox regression (Table 2). The significant pre-transplantation predictive factors identified were then introduced by *forward conditional* method in a multivariate Cox model (Table 3). Donor age, cold-ischemia time, HLA-DR mismatches ≥1, and the presence of DSA remained independent predictive variables in the multivariate analysis (Table 3, A). The adjusted-risk of allograft failure was more than double in recipients with DSA at time of transplantation (HR 2.133; CI 95% 1.379–3.300; *p* = 0.001). However, when the DSA population was subsequently stratified according to the DSA C1q-binding ability (Table 3, B), only the subset of patients with preformed C1q-binding DSA showed a significantly increased adjusted-risk of allograft loss compared to patients without DSA (HR 4.012; CI 95% 2.326–6.919; *p* < 0.001). Interestingly, we did not find significant differences at any point throughout the follow-up time between patients with preformed non-C1q-binding DSA and patients without DSA, regarding allograft loss adjusted-risk (HR 1.389; CI 95% 0.784–2.461; *p* = 0.260).

**Table 2:** Pre-transplantation clinical and immunological risk factors associated with allograft loss.

Factor	No. of patients	Hazard ratio(s) (HR)	CI 95%	*p*
**Donor age (per 1 year of increment)**	389	1.016	1.003–1.030	0.015
**Cold-ischemia time (per 1 h of increment)**	389	1.055	1.028–1.082	<0.001
**Recipient age (per 1 year of increment)**	389	0.993	0.978–1.008	0.349
**Sex female**				
No	248	1.00		
Yes	141	1.475	0.975–2.232	0.066
**Re-transplantation**				
No	332	1.00		
Yes	57	2.259	1.407–3.626	0.001
**Time on waiting list (per 1 year of increment)**	389	1.025	0.987–1.064	0.200
**Human leukocyte antigen (HLA)-A, -B mismatches (per 1 mismatch of increment)**	389	1.143	0.927–1.409	0.211
**HLA-DR mismatches ≥1**				
No	119	1.00		
Yes	270	1.929	1.152–3.232	0.013
**Anti-calcineurin drugs**				
Tacrolimus	215	1.00		
Cyclosporine	174	0.962	0.636–1.456	0.856
**Triple maintenance immunosuppressant therapy**				0.219
Calcineurine inhibitor + MMF + Pred	263	1.00		–
Calcineurine inhibitor + Aza + Pred	104	1.133	0.720–1.785	0.589
Calcineurine inhibitor + Rapamycin + Pred	22	0.759	0.276–2.088	0.593
**Induction therapy^a^**				
No	347	1.00		
Yes	42	1.580	0.878–2.843	0.127
**cPRA^b^**	389	1.010	1.005–1.015	<0.001
**Presence of DSA**				
No	297	1.00		
Yes	92	2.009	1.306–3.091	0.002
**Presence of DSA and C1q-binding ability**				
No-DSA	297	1.00	–	–
Non-C1q-binding DSA	62	1.270	0.720–2.238	0.409
C1q-binding DSA	30	4.160	2.420–7.151	<0.001

*Univariate Cox analysis*.

*CI, confidence interval; MMF, mycophenolate mofetil, Pred, prednisone; Aza, azathioprine*.

*^a^Induction treatment consisted of thymoglobulin*.

*^b^Calculated panel reactive antibody (cPRA) value at time of transplantation, retrospectively calculated according to unacceptable antigens detected by SAB-panIgG assay using OPTN database*.

**Table 3:** Risk allograft-loss assignment according to the presence of DSA at time of transplantation (A) and the DSA C1q-binding ability (B) after the adjustment for other clinical and immunological pre-transplantation predictive factors including donor age, cold-ischemia time, and human leukocyte antigen (HLA)-DR mismatches.

Multivariate Cox regression^a^	No. of patients	Hazard ratio(s) (HR)	CI 95%	*p*
**Donor age (per 1 year of increment)**	389	1.016	1.003–1.029	0.014
**Cold-ischemia time (per 1 h of increment)**	389	1.054	1.028–1.082	<0.001
**HLA-DR mismatches ≥1**				
No	119	1.00	–	–
Yes	270	1.896	1.129–3.851	0.016

**Model A**				
**Presence of DSA (at time of transplantation)**				
No	297	1.00		
Yes	92	2.133	1.379–3.300	0.001

**Model B**				
**Presence of DSA and C1q-binding ability**				
No-DSA	297	1.00		
Non-C1q-binding DSA	62	1.389	0.784–2.461	0.260
C1q-binding DSA	30	4.012	2.326–6.919	<0.001

*Multivariate model by Cox regression*.

*^a^The significant predictive factors in the univariate analysis were introduced by forward conditional method in the multivariate analysis*.

*CI, confidence interval*.

Both multivariate predictive models were explored by receiver operator characteristic analysis. AUC was 0.704 (CI 95%, 0.645–0.763) for the conventional predictive model based on the presence of DSA detected by the standard SAB-panIgG assay (Table 3, A). The AUC of the model which included the DSA C1q-binding ability (Table 3, B) became enhanced (AUC = 0.725; CI 95%, 0.665–0.782). In addition, we explored the predictive value of a multivariate model considering the presence of high-MFI DSA (MFI ≥10,000) at time of transplantation (Table S1 in Supplementary Material). The AUC of this model was lower than the model based on the presence of C1q-binding DSA (AUC = 0.711; CI 95%, 0.652–0.770).

### Antibody MFI Value and C1q Reactivity

**Figure 3:**
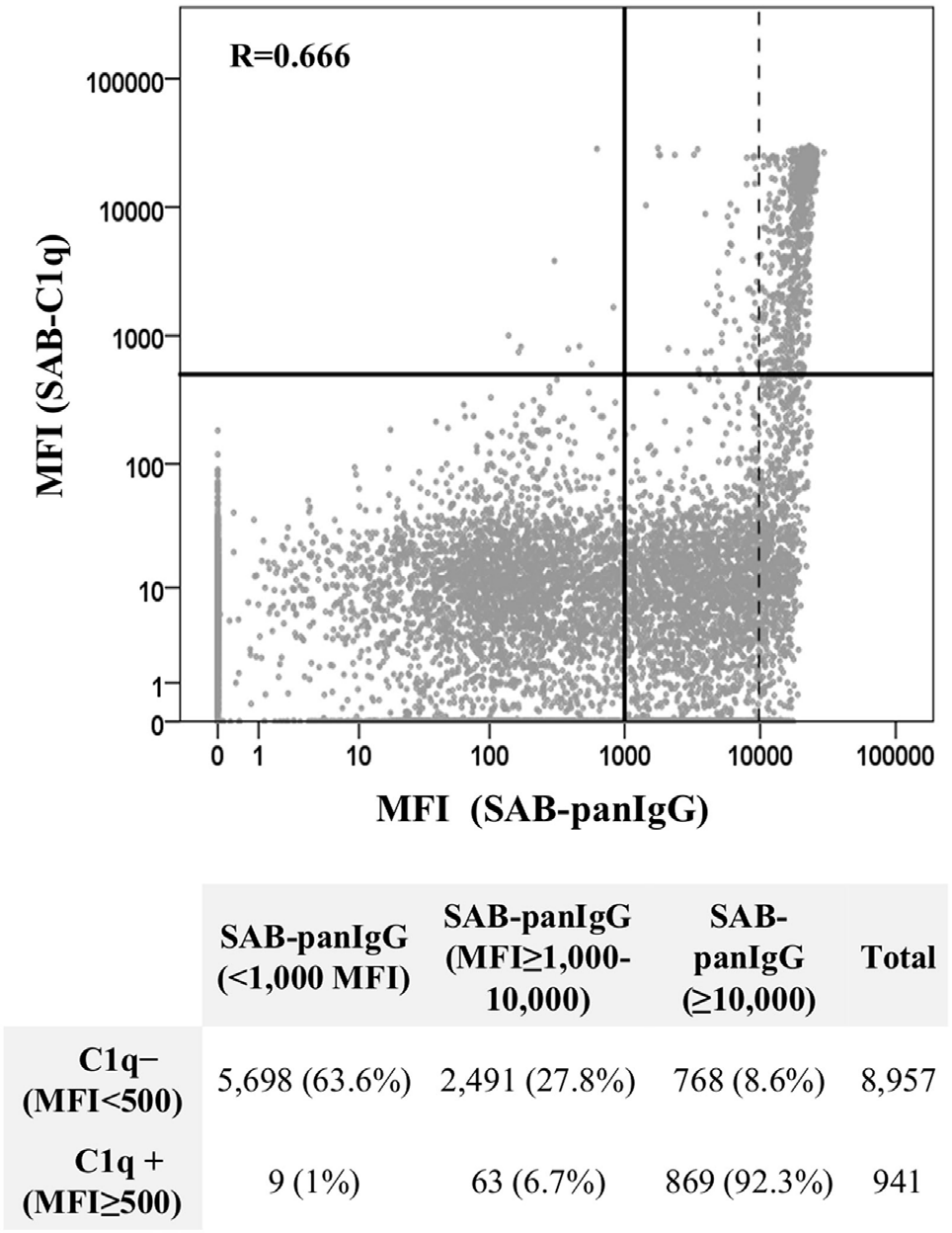
Correlation between SAB-panIgG mean fluorescence intensity (MFI) values and SAB-C1q MFI values. MFI values of single Luminex beads were plotted in a log-scale scatter graph. Table under the graph shows the number of negative (MFI <500) and positive (MFI ≥500) beads in SAB-C1q assay according to their SAB-panIgG MFI value.

A total of 9,898 data points, from neat-serum sample analyses belonging to the 92 patients with preformed DSA and representing single Luminex beads, were displayed in a log-scale scatter plot according to their MFI value (baseline) obtained by SAB-panIgG assay and their respective MFI value obtained by SAB-C1q assay (Figure 3). Among 4,191 positive beads detected by SAB-panIgG, 932 (22.2%) were also positive in SAB-C1q assay, whereas 3,259 (77.8%) tested negative. Nine out of 941 positive beads in SAB-C1q assay were not detectable as positive in the standard SAB-panIgG assay. The MFI average of positive antibodies capable of binding C1q was significantly higher than that of positive antibodies incapable of binding C1q (18,816 vs. 6,495; *p* < 0.001). Among 941 C1q-binding antibodies, 869 (92.3%) showed a neat-serum MFI value of ≥10,000 in the standard SAB-panIgG assay and only 72 of them (7.7%) showed a neat-serum MFI value below 10,000. The correlation between MFI values of each bead obtained using both tests was of 0.666 (Pearson’s correlation). In addition, the correlation between the presence of antibodies with high-MFI value (MFI ≥10,000) in SAB-panIgG assay and their positivity in SAB-C1q assay (MFI ≥500) was of 0.661 (Pearson’s correlation).

## Discussion

In this retrospective study, 389 single-kidney transplanted patients were carefully characterized according to the presence of preformed DSA and the DSA C1q-binding ability, using SAB-panIgG and SAB-C1q assays as antibody detection tests. Our results showed that patients with preformed C1q-binding DSA had worse 7-year allograft survival than patients with non-C1q-binding DSA or without DSA. Interestingly, among the high-MFI DSA population, those patients whose DSA were further capable of binding C1q showed a poorer outcome. Moreover, in our multivariate predictive model for assessing the risk of allograft loss, only the presence of the C1q-binding DSA subset remained an independent predictor after stratifying the DSA population according to the C1q-binding ability and adjusting the model for other pre-transplantation predictive factors, including donor age, cold-ischemia time, and HLA-DR mismatches.

Since the development of highly sensitive solid-phase tests, the presence of DSA detected by SAB-panIgG assay, under a negative CDC cross-match context, has been associated with poor transplant prognoses (22–24). Consistent with these findings, our results indicate that the presence of preformed DSA increases the risk of allograft loss, supporting the theory that these antibodies damage the allograft. In addition, our study also reveals that not all preformed DSA detected by SAB-panIgG assay are equally pathogenic, suggesting that the significant injury on allograft occurs mainly when preformed C1q-binding DSA are present.

The role of DSA according to the C1-binding ability has previously been evaluated, remaining one of the main investigation lines of humoral response against transplanted allografts. Several studies have demonstrated that the *de novo* appearance of C1q-binding DSA after transplantation is strongly associated with worse allograft outcome. Loupy et al. (25) reported that the presence of C1q-binding DSA was associated with an increased rate of AMR, more severe graft injury phenotypes and an increased risk of allograft loss. Previously, Sutherland et al. (26) had already demonstrated the greater risk of allograft loss of *de novo* C1q-binding DSA. In the same line, Piazza et al. (27) showed that the presence of C1q-binding, but not non-C1q-binding *de novo* DSA was a biomarker of worse transplant outcome. More recent studies have found that the development of AMR and the subsequent allograft injury occurs mainly in the presence of C1q-binding DSA (28, 29). However, despite the growing evidence about the harmful role that C1q-binding DSA play on transplant outcome, supported by the theoretical higher ability of these antibodies to activate the *in vivo* complement cascade, the clinical usefulness of SAB-C1q assay in the pre-transplantation allograft allocation has not been accurately determined.

Authors analyzing the clinical relevance of the pre-transplant C1q-binding ability have reported controversial results. While initial studies evaluating the usefulness of SAB-C1q assay in heart-transplanted recipients showed a strong association between preformed C1q-binding DSA and the risk of AMR and premature allograft loss (19, 20), subsequent studies discussed its clinical use for allograft allocation. Otten et al. (30) could not assess the clinical significance of C1q-binding DSA regarding allograft survival due to the low prevalence of sera scoring DSA-positive in the SAB-C1q assay. Crespo et al. (31), and more recently, Thammanichanond et al. (32) in small cohorts of patients with DSA (28 and 48, respectively) did not find any association between allograft outcome and the presence of C1q-binding or non-C1q-binding DSA, suggesting a limited predictive value for SAB-C1q assay. However, these studies did not provide any data about the theoretically pathological role caused by non-C1q-binding DSA with regard to a control population without DSA.

The present report, evaluating 92 transplanted patients with preformed DSA, demonstrates that the severe effect on allograft function is caused when preformed DSA are able to bind complement and provides evidence of their limited impact when they are non-C1q-binding DSA. Interestingly, SAB-C1q assay allowed us to define groups of patients with different allograft survival among those with preformed high-MFI DSA (Figure 2B), whereas allograft survival of non-C1q-binding DSA recipients was similar (*p* = 0.457) regardless of the MFI value (Figure S3 in Supplementary Material). Taken together, these data suggest that the neat-serum MFI value alone, which only offers a semi-quantitative measured of antibody level at best (33), is not entirely reliable for predicting transplant outcome, thus other antibody properties, such as the C1q-binding ability, should be considered. Moreover, our results support recent findings showing that SAB-C1q assay improves the AMR prediction with regard to the MFI value (34), assuming the strong association between AMR and allograft failure (35). The use of SAB-C1q assay within a sensitized population could provide added value to the conventional immunological risk stratification based on the MFI value of DSA.

Not surprisingly, the C1q-binding ability was a characteristic mainly found in beads showing high MFI values in the standard SAB-panIgG assay (Figure 3). In this regard, other authors have already shown that the ability to bind C1q is linked to the antibody strength (20, 28). However, this association is far from perfect when the antibody strength is assigned using the neat-serum MFI value and either low-MFI but C1q-binding antibodies or high-MFI but non-C1q-binding antibodies may be detected, as depicted in Figure 3. The detection of low-MFI antibodies capable of binding C1q is commonly a consequence of a prozone effect, a phenomenon that hides the real strength of antibodies (20). The treatment of neat-samples with EDTA or dithiothreitol may somewhat eliminate this inhibitory effect (36), which would enhance the relationship between the antibody C1q-binding ability and its MFI value. Conversely, high-MFI antibodies incapable of binding C1q could denote low antibody strength, as reported by Tambur et al. (37), who using serum serial dilutions for anti-HLA antibody detection provided a more reliable estimation of their real strength (titer) and demonstrated a strong association between low antibody titers and the inability to bind C1q. The SAB-C1q assay, as well as titration studies, enables the real strength of antibodies to be unmasked.

Otherwise, given the different avidity for the C1q protein complex exhibited by the four IgG subclasses (38), the IgG isotype pattern of a particular antibody is determinant for its potential C1q-binding ability. Until now, IgG subclass studies showed that anti-HLA antibodies are not usually comprised of a unique IgG subclass but of a variable mixture of them, IgG1 being by far the most common (39–42). Arguably, the direct correlation between the antibody real strength (titer) detected by SAB-panIgG assay and their C1q-binding ability could be largely explained by the high prevalence of IgG1, as the immunodominant subclass. Emerging evidence supports that the differences between C1q- and non-C1q-binding antibodies are not usually due to the quality but to the quantity (titer) of the IgG subclasses comprising them (20, 28, 37, 41, 42). Indeed, a negative SAB-C1q assay result does not mean that the antibody investigated was composed of isotypes without the biological capacity to activate the complement, as would be expected, but that it may contain a certain amount of strong C1q-binding IgG subclasses (41).

Only in the form of hexamers *via* Fc:Fc non-covalent interactions, IgG is endowed to bind the C1q component and assemble C1q:(IgG)_6_ complexes (43, 44). This status implies that a critical antibody density bound to its target antigen is needed to provide a sufficiently avid C1q-binding site, what supports the close relationship between C1q-binding ability and antibody strength (titer). A low titer of specific IgG subclasses (IgG1/IgG3) comprising a particular anti-HLA antibody would not be enough to conform the hexameric complexes on the antigens of Luminex beads, preventing the efficient recruitment of C1q and its subsequent detection by SAB-C1q assay. All evidence described seems to indicate that the strength (titer) of strong C1q-binding subclasses, mainly IgG1, is the major limiting factor for the anti-HLA antibody C1q-binding status assignment. Considering the low prevalence of isolated IgG2 and/or IgG4 subclasses described in previous reports, the *per se* anti-HLA antibody inability to bind C1q seems to be uncommon (39, 41, 42).

The kinetics of IgG hexamerization is a concentration-dependent dynamic process (44). Thus, we hypothesize that the lower impact on allograft outcome of non-C1q-binding DSA observed in our cohort could be a consequence of the low titer of strong C1q-binding isotypes comprising them, which would not reach the critical threshold to efficiently recruit the C1q protein and trigger the complement cascade *in vivo*, the major pathway of antibody-mediated injury (15). Similarly, the absence of strong C1q-binding subclasses in a low proportion of non-C1q-binding antibodies could also limit their harmful impact on the allograft. The characterization of DSA as non-C1q-binding antibodies is not strictly associated with low antibody strength, because a high level of non-C1q-binding subclasses may be present. This fact explains the not completely perfect correlation between the C1q-binding ability and the antibody titer and suggests that the real strength (titer) does not provide exactly the same information as the ability to bind C1q. Both properties (titer and C1q-binding ability) should be integrated in future analyses to provide more valuable insights into the assessment of the immunological risk of anti-HLA antibodies.

It is well established that DSA are responsible for allograft damage through a wide spectrum of effector functions, which range from complement activation to FcγR-dependent macrophage and NK cell functions (45). Recent findings have associated the presence of circulating DSA-IgG4 with subclinical AMR and later allograft injury characterized by a predominance of chronic histological features (42), which supports that even in the absence of complement activation, antibodies may lead to non-complement-mediated chronic allograft damage (46). From this perspective, all DSA, regardless of their C1q-binding ability, should ideally be avoided, but unfortunately, this is not a plausible option for an increasing proportion of highly sensitized patients. Developing desensitization protocols to reduce the incidence of rejection and maintain low levels of antibodies for long periods of time, optimizing the allograft exchange programs and improving our understanding of the pathogenicity of antibodies are future challenges to ensure the success of transplantation in highly sensitized patients. Since our study identifies different groups of risk based on the C1q-binding ability of DSA, we postulate a new pre-transplantation and enhanced stratification algorithm. This new algorithm should be cautiously interpreted and should be particularly addressed to those patients whose transplantation possibilities are considerably limited.

Our study has limitations. First, it is observational and consequently does not provide complete information about the damaging pathways caused by anti-HLA antibodies, given the close relationship between preformed DSA and AMR revealed in different organ transplants by histopathological findings and functional manifestations (47). Since diagnostic criteria for allograft rejection varied over the time course of the study, the association between AMR and the C1q-binding ability could not be accurately ascertained. Second, we could not avoid the heterogeneity in the immunosuppressant treatments implemented, although this factor did not have a significant predictive value. Third, the non-inclusion of the presence of antibodies against HLA-Cw and -DP antigens could be a possible confounding factor in a proportion of patients, assuming their clinical relevance for allograft outcome (48). Finally, when we stratified the study population according to the DSA C1q-binding status, the prevalence of patients with preformed C1q-binding DSA was low within the population analyzed. However, sample size was enough to find differences within the DSA group.

In conclusion, our report demonstrates that only preformed C1q-binding DSA represent a total contraindication to kidney transplantation. The clinical use of SAB-C1q assay for the identification of unacceptable mismatches would permit us to better stratify the risk of allograft loss. This new algorithm might increase the limited allograft allocation of highly sensitized patients, predefined by the standard SAB-panIgG assay, shortening the waiting time for these patients, many of them with poor prognosis due to severe associated clinical conditions.

## Ethics Statement

This study was approved by the Ethics Committee of the Reina Sofia University Hospital (ref. 2465).

## Author Contributions

RS and JM performed the study design. M-LA, CR-H, and CA provided and acquired clinical data. JM and AN conducted the laboratory analysis and interpreted the results. JM performed the statistical analysis. JM and AN wrote the draft. AR-B, PA, and RS reviewed the final version. All authors: provided intellectual content, contributed to the article writing, and approved the final version.

## Conflict of Interest Statement

The authors declare that the research was conducted in the absence of any commercial or financial relationships that could be construed as a potential conflict of interest.

## Abbreviations

AMR, antibody-mediated rejection; AUC, area under the curve; CDC, complement-dependent cytotoxicity; CI, confidence interval; DSA, donor-specific anti-HLA antibody; HLA, human leukocyte antigen; HR, hazard ratio(s); MFI, mean fluorescence intensity; PRA, panel reactive antibody; SAB, single antigen beads; VXM, virtual cross-match.
