# Supplementary material for: Impact of Preformed Donor-Specific Anti-Human Leukocyte Antigen Antibody C1q-Binding Ability on Kidney Allograft Outcome
Source: Front Immunol. 2017 Oct 31;8:1310. doi: 10.3389/fimmu.2017.01310 (PMC5671504; doi:10.3389/fimmu.2017.01310)
Supplement: Supplementary file 1 [file table_1.docx]

Supplementary Material

Impact of Preformed Donor-specific anti-HLA Antibody C1q-binding Ability on Kidney Allograft Outcome

**Juan Molina^1^, Ana Navas^1, *^, María-Luisa Agüera^1, 2^, Cristian Rodelo-Haad^1^, Corona Alonso^1, 3^, Alberto Rodríguez-Benot^1, 2^, Pedro Aljama^1, 2^, Rafael Solana^1, 4^**

^1^Maimonides Biomedical Research Institute of Cordoba (IMIBIC)/Reina Sofia University Hospital/University of Cordoba, Spain

^2^Department of Nephrology, Reina Sofia University Hospital, Cordoba, Spain

^3^Department of Allergy and Immunology, Reina Sofia University Hospital, Cordoba, Spain

^4^Department of Immunology, Infanta Cristina University Hospital, Badajoz, Spain

***Correspondence**:

Ana Navas

[ananavasromo@gmail.com](mailto:ananavasromo@gmail.com)

# Supplementary Figures and Tables

## Supplementary Tables

**Table S1.** Risk allograft-loss assignment according to the presence of high-MFI value DSA (≥10,000) at time of transplantation after the adjustment for other clinical and immunological pre-transplantation predictive factors including: donor age, cold ischemia time and HLA-DR mismatches. Multivariate model by Cox regression.

| **Multivariate Cox regression** | **No. of patients** | **Hazard**  **ratio (HR)** | **CI^a^ 95%** | **p** |
| --- | --- | --- | --- | --- |
| **Donor age (per 1 year of increment)** | 389 | 1.015 | 1.003-1.028 | 0.019 |
| **Cold ischemia-time (per 1 hour of increment)** | 389 | 1.055 | 1.028-1.082 | <0.001 |
| **HLA-DR mismatches ≥ 1** |  |  |  |  |
| No | 119 | 1.00 | -- | -- |
| Yes | 270 | 1.873 | 1.113-3.150 | 0.018 |
| **Presence of high-MFI value DSA (at time of transplantation)** |  |  |  |  |
| No | 343 | 1.00 | -- | -- |
| Yes | 46 | 2.724 | 1.665-4.456 | <0.001 |

^a^CI denotes confidence interval
