# Supplementary material for: Impact of Preformed Donor-Specific Anti-Human Leukocyte Antigen Antibody C1q-Binding Ability on Kidney Allograft Outcome
Source: Front Immunol. 2017 Oct 31;8:1310. doi: 10.3389/fimmu.2017.01310 (PMC5671504; doi:10.3389/fimmu.2017.01310)
Supplement: Supplementary file 2 [file image_1.pdf]

Supplementary Material

Impact of Preformed Donor-specific anti-HLA Antibody C1q-binding Ability on Kidney Allograft Outcome

Juan Molina[1], Ana Navas[1, *], María-Luisa Agüera[1, 2], Cristian Rodelo-Haad[1], Corona Alonso[1, 3], Alberto Rodríguez-Benot[1, 2], Pedro Aljama[1, 2], Rafael Solana[1, 4]

[1]Maimonides Biomedical Research Institute of Cordoba (IMIBIC)/Reina Sofia University Hospital/University of Cordoba, Spain

[2]Department of Nephrology, Reina Sofia University Hospital, Cordoba, Spain

[3]Department of Allergy and Immunology, Reina Sofia University Hospital, Cordoba, Spain

[4]Department of Immunology, Infanta Cristina University Hospital, Badajoz, Spain

***Correspondence**:

Ana Navas

ananavasromo@gmail.com

1 Supplementary Figures and Tables

1.1 Supplementary Figures

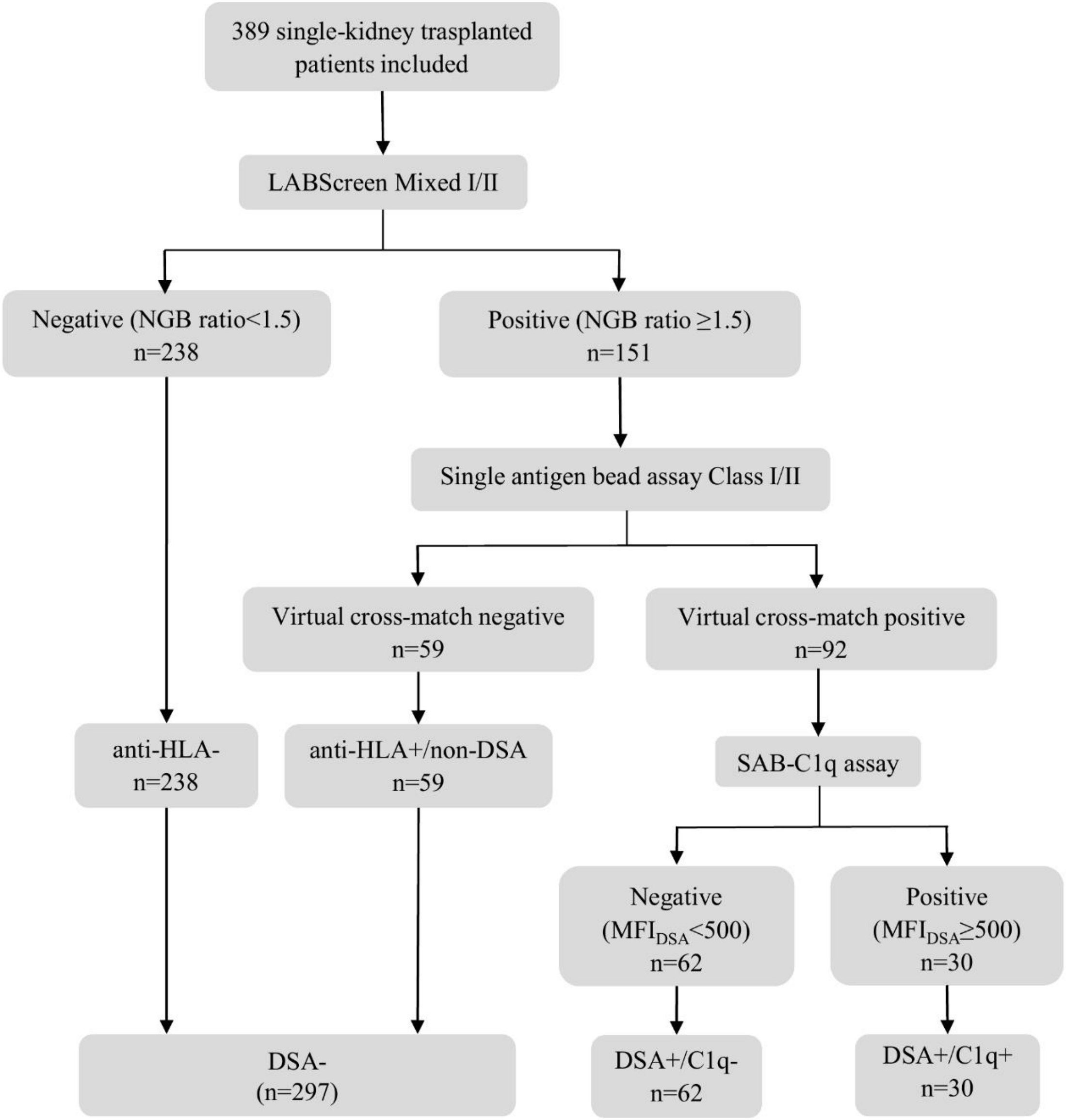

Figure S.1. Methodology of the study. Serum samples were retrospectively analyzed using the Luminex Mixed Screen assay. When normalized background ratio (NGB) was greater than 1.5, samples were analyzed using single antigen bead pan-IgG assay. Then, a virtual cross-match was performed using the information on donor HLA typing. Finally, when the virtual cross-match was positive, serum samples were analyzed by single antigen bead (SAB)-C1q assay.
