# Supplementary material for: Impact of Preformed Donor-Specific Anti-Human Leukocyte Antigen Antibody C1q-Binding Ability on Kidney Allograft Outcome
Source: Front Immunol. 2017 Oct 31;8:1310. doi: 10.3389/fimmu.2017.01310 (PMC5671504; doi:10.3389/fimmu.2017.01310)
Supplement: Supplementary file 3 [file image_2.pdf]

Supplementary Material

Impact of Preformed Donor-specific anti-HLA Antibody C1q-binding Ability on Kidney Allograft Outcome

Juan Molina[1], Ana Navas[1, *], María-Luisa Agüera[1, 2], Cristian Rodelo-Haad[1], Corona Alonso[1, 3], Alberto Rodríguez-Benot[1, 2], Pedro Aljama[1, 2], Rafael Solana[1, 4]

[1]Maimonides Biomedical Research Institute of Cordoba (IMIBIC)/Reina Sofia University Hospital/University of Cordoba, Spain

[2]Department of Nephrology, Reina Sofia University Hospital, Cordoba, Spain

[3]Department of Allergy and Immunology, Reina Sofia University Hospital, Cordoba, Spain

[4]Department of Immunology, Infanta Cristina University Hospital, Badajoz, Spain

***Correspondence**:

Ana Navas

ananavasromo@gmail.com

1 Supplementary Figures and Tables

1.1 Supplementary Figures

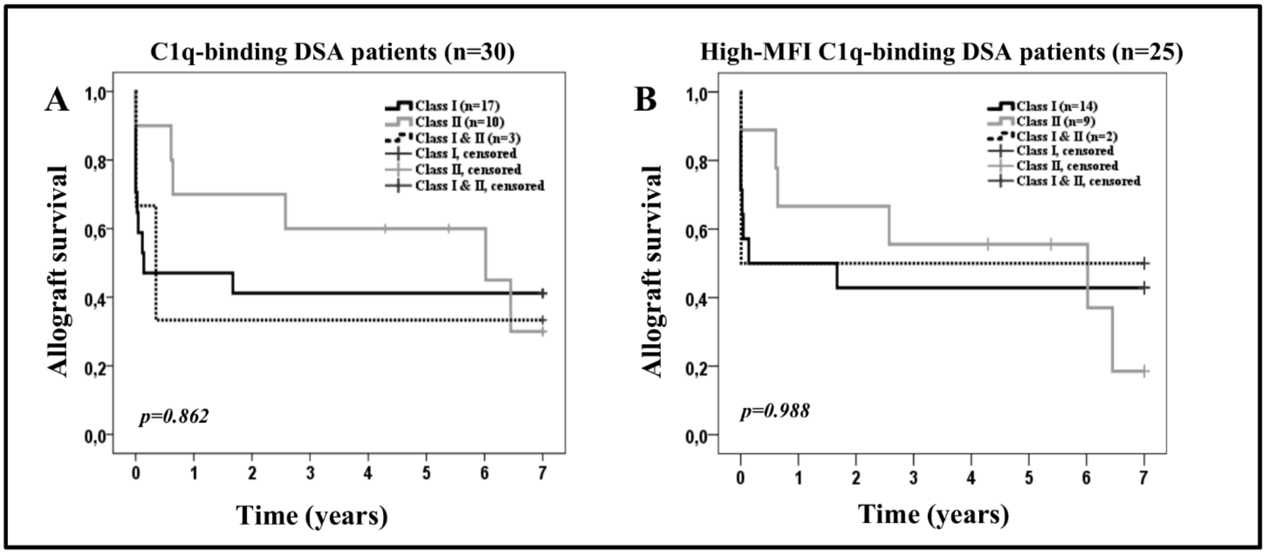

Figure S.2. Kaplan-Meier curves for kidney allograft survival up to 7-years in 30 patients with C1q-binding DSA **(A)** and 25 patients with high-MFI C1q-binding DSA **(B)** stratified according to the HLA Class molecule (Class I and/or II) against which C1q-binding donor-specific anti-HLA antibodies (C1q-binding DSA) were directed. Curves were compared using the log-rank test.
