# Supplementary material for: Impact of Preformed Donor-Specific Anti-Human Leukocyte Antigen Antibody C1q-Binding Ability on Kidney Allograft Outcome
Source: Front Immunol. 2017 Oct 31;8:1310. doi: 10.3389/fimmu.2017.01310 (PMC5671504; doi:10.3389/fimmu.2017.01310)
Supplement: Supplementary file 4 [file image_3.pdf]

Supplementary Material

Impact of Preformed Donor-specific anti-HLA Antibody C1q-binding Ability on Kidney Allograft Outcome

Juan Molina[1], Ana Navas[1, *], María-Luisa Agüera[1, 2], Cristian Rodelo-Haad[1], Corona Alonso[1, 3], Alberto Rodríguez-Benot[1, 2], Pedro Aljama[1, 2], Rafael Solana[1, 4]

[1]Maimonides Biomedical Research Institute of Cordoba (IMIBIC)/Reina Sofia University Hospital/University of Cordoba, Spain

[2]Department of Nephrology, Reina Sofia University Hospital, Cordoba, Spain

[3]Department of Allergy and Immunology, Reina Sofia University Hospital, Cordoba, Spain

[4]Department of Immunology, Infanta Cristina University Hospital, Badajoz, Spain

***Correspondence**:

Ana Navas

ananavasromo@gmail.com

1 Supplementary Figures and Tables

1.1 Supplementary Figures

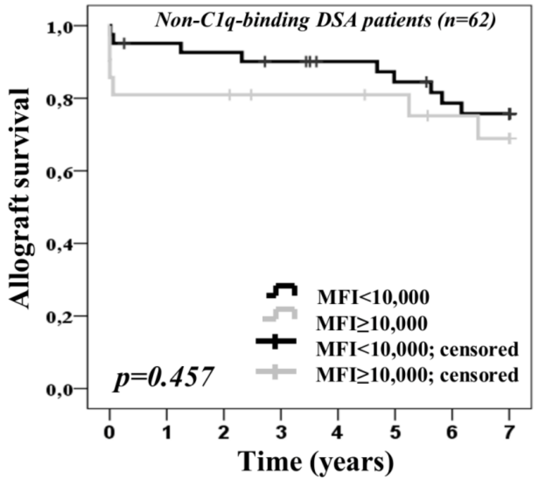

No. of remaining cases and no. of cumulative events

	0	1	2	3	4	5	6	7
**MFI<10,000**	41;	38;	37;	35;	32;	30;	27;	26;
**n=41**	0	2	3	4	4	6	8	9
**MFI≥10,000**	19;	17;	17;	15;	15;	14;	12;	11;
**n=21**	2	4	4	4	4	4	5	6

Figure S.3. Kaplan-Meier curves for allograft survival up to 7-years of the 62 patients with preformed non-C1q-binding donor-specific anti-HLA antibodies (non-C1q-binding DSA) stratified according to DSA MFI value at time of transplantation. The DSA were categorized setting a MFI cut-off of 10,000. Curves were compared using the log-rank test.
